# Physical Strength Perception of Older Caregivers in Rural Areas

**DOI:** 10.3390/medicina55100692

**Published:** 2019-10-16

**Authors:** Laura Muñoz-Bermejo, Santos Villafaina, Daniel Collado-Mateo, Salvador Postigo-Mota, José Carmelo Adsuar

**Affiliations:** 1 University Center of Mérida, University of Extremadura, 06800 Mérida, Spain; lauramunoz@unex.es; 2 Faculty of Sport Science, University of Extremadura, 10003 Cáceres, Spain; jadssal@unex.es; 3 Centre for Sport Studies, Rey Juan Carlos University, 28943 Fuenlabrada, Madrid, Spain; 4 Faculty of Medicine, University of Extremadura, 06006 Badajoz, Spain; spostigo@unex.es

**Keywords:** caregivers, aging, muscle strength, older adults, independence

## Abstract

*Background and objective*: In an aging population, it is increasingly common for older adults to take care of other older adults. Caregiving tasks may be conditioned by the aging process. This study aims to analyze the perceived physical strength of older caregivers and its impact on the functional capacity to engage in caregiving activities. *Methods*: A discretionary sampling of caregivers (*N* = 107), ≥65 years old, in the rural health area of Badajoz (Spain) participated in this cross-sectional study. Measurements included questions about the caregiver’s role (experience, years, hours, difficulties, demands) and their perceived physical strength, ability to perform activities of daily living (ADL), need for help or difficulty for caring. *Results*: Older caregivers from rural areas perceived a lack of physical strength (71%). These caregivers need more help, have more difficulties, and show less ability to perform ADL. Furthermore, around 80% of the people who had a lack of strength were caring for people with severe or total dependence. There is a direct correlation between the perceived lack of physical strength and the ability to perform basic (r = 0.382, *p* < 0.01) and instrumental (r = 0.370, *p* < 0.01) activities. *Conclusions*: Therefore, the perception of strength and the characteristics of the cared for person may be crucial variables to successfully conduct caregiving tasks.

## 1. Introduction

The increase in the population’s life expectancy has increased the demand for older adult care in western industrialized countries [1]. A new challenge has been presented to health systems that coincides with profound changes in the social, family, and caregiver structure [2]. Usually, the care demands fall upon informal caregivers, i.e., the family, which is the primary health care provider [3]. In this regard, these chores are commonly assumed by kinships’ older adults, such as the wife or husband, siblings, and even children exceeding the chronological barrier of 60 years [4]. Thus, informal care is mostly assumed by older parents (32%) and spouses (36%) [5].

According to the Informal Support Survey for older adults in Spain, more than 30% of older caregivers are over the age of 65 [6]. The caregiving role in older people can lead to poor health outcomes [7,8] with familial, social, economic, and spiritual consequences [9]. Moreover, older caregivers are more affected by physical burden [10], demand more health services [11], and receive less social support [12]. Furthermore, the development or worsening of health problems is associated with increased susceptibility to these burdens [13]. For these reasons, older caregivers report greater dependence on instrumental activities of daily living (IADL) [14] and greater morbidity associated with physical frailty [15], which implies a decrease in the quality of life related to health [16] and satisfaction with life [17].

The decrease in age-related muscle mass is an important health problem and has a negative influence on the ability to perform daily life tasks, which leads to a reduced quality of life [18]. Caregivers are also aware of their weakened strength and health problems caused by aging, which diminishe their ability to provide care, increase their efforts to assist, and make them feel more tired [19]. Therefore, older caregivers present a diminished capacity and dedication to develop caregiving tasks [4,20].

There are many sources of care for people with long-term chronic illnesses, and there are specific directives for their caregivers. To obtain excellence in the care of people suffering from chronic diseases, the focus of care should be on the caregiver [2]. It is important that resources and strategies are offered so that the caregiver can manage daily challenges in full support of physical safety and activity performance [21,22,23].

Therefore, the main aim of the present study was to evaluate the perceived lack of strength among older caregivers in rural areas. Moreover, we aimed to identify gender differences and the influence of the perceived lack of strength in care-related variables, such as difficulties, need for help, or characteristics of the care recipient.

## 2. Materials and Methods

### 2.1. Participants

A total of 107 participants (*N* = 107; with a median ± IQR of 78 ± 8 years) were included in this cross-sectional study. Regarding gender, the participants included 64 women (59.8%) and 43 men (40.2%). All participants gave written consent to participate in the study. Procedures were approved by the University Research Ethics Committee (approval number: 212/2019 from 8 October 2019).

Inclusion criteria included:

(1) Living in a rural town (fewer than 10,000 inhabitants);
(2) Being older than, or equal to, 65 years old;
(3) Being the primary caregiver of a dependent adult older than or equal to 65 years old. We considered primary caregivers those who assume responsibility for nursing and provide the highest amount of care;
(4) Being able to give written informed consent.

### 2.2. Procedure

Data were collected from each caregiver through an individual interview that was performed in each caregiver´s home. The interview was arranged with the support of the Social Work Service of the Health Centers.

The Caregivers Questionnaire, included in the Disability, Personal Autonomy, and Dependency Situations Survey (AGE 2008) [24] was used as a reference. In this regard, the caregivers´ perception of physical strength was assessed through item number 15 of the Caregivers Questionnaire of the National Statistics Institute. This dichotomous item referred to the “perception of special difficulty due to the lack of physical strength”.

Moreover, the Barthel Index [25], consisting of ten activities graded according to the patient´s level of dependence, was used to assess the ability to perform the primary activities of daily living (ADL). These activities include: eating (score 5 or 10); bathing/washing (score 0 or 5); dressing (score 5 or 10) grooming (score 0 or 5); deposition (score 5 or 10); urinating (score 5 or 10); toileting (score 5 or 10); transfers chair/bed (score 5, 10, or 15); mobility (score 5, 10, or 15); and going up and down stairs (score 5 or 10). The sum of each ADL score is the Barthel Index total score, which can range from 0 to 100. The higher the score, the more independent the patient. The Barthel Index presents inter-observer reliability between 0.47 and 1.00, and intra-observer reliability between 0.84 and 0.97 [26,27]. The internal consistency corresponds to a Cronbach´s alpha of 0.86–0.92 [28] in the original version.

The Lawton and Brody index [29] was used to determine the capacity to perform IADL. This index includes eight activities (ability to use the telephone, shopping, meal preparation, housekeeping, laundry, use of transport, responsibility for medication, and handling financial matters). Each area scores a maximum of 1 and a minimum of 0 points. Thus, maximum dependence would be considered 0 points, whereas total independence corresponds to 8 points. The ranking categories include 0–1: total dependency; 2–3: severe dependency; 4–5: moderate dependency; 6–7: slight dependency; and 8: independence. The inter-observer reliability for this questionnaire was 0.85.

The Zarit Burden Inventory scale [30] was also used to identify the caregivers´ overload. This is an instrument used to quantify the degree of overload suffered by caregivers of dependent adults. The Spanish version has 22 questions in a Likert format. For each item, the caregiver indicates how often they feel a certain way, using a scale of 1 (never), 2 (rarely), 3 (sometimes), 4 (quite a few times), and 5 (almost always). The scores obtained in each item were summarized, and the final score represented the overload degree of the caregiver. Its internal consistency was high, with a Cronbach alpha coefficient of 0.91.

Lastly, the Duke-UNC-11 Functional Social Support Questionnaire [31] was used to determine the perceived social support of older caregivers. This scale has 11 items in a Likert response scale of 1 (“much less than I want”) to 5 (“as much as I want”). The scoring ranges between 11 and 55 points. In the Spanish version, a cut-off point was chosen at the 15th percentile, corresponding to a score of <32. A score equal to or greater than 32 indicates standard support, while less than 32 points indicates perceived low social support. In the Spanish population, the internal consistency was 0.90 [32].

### 2.3. Statistical Analysis

In order to describe the total sample of caregivers, the prevalence of the categorical variables and the mean with standard deviation, the median, interquartile range (IR), and the maximum and minimum values (range) for the quantitative variables were calculated.

In light of the results of the Kolmogorov–Smirnov and Shapiro–Wilk tests, the data were analyzed with non-parametric methods. The Mann–Whitney U test or Chi-Square test was applied to evaluate between-gender differences. Furthermore, these tests were also used to compare those caregivers with a lack of strength with those who did not perceive they had a lack of strength. Hypothesis contrasts were considered significant when *p* < 0.05.

The correlation coefficient of Spearman ρ (rho) was used to measure the association between variables. Hypothesis contrasts were considered significant when *p* < 0.05. The statistical analysis was performed using SPSS version 22.0© software for Windows.

## 3. Results

Table 1 shows the sociodemographic variables and characteristics of the total sample and both women and men caregivers. Statistically significant differences (*p* < 0.05) between genders were found for age, previous caregiving experience, doubts about better care, the gender of the person being cared for, and the ability to develop IADL.

Table 2 shows the differences between those participants who reported a perception of inadequate strength levels and those who perceived they did not have a lack of strength. Age was slightly different between groups, and those who were older showed a higher perceived lack of strength but did not reach the statistically significance level (*p* = 0.051).

Clear between-group differences were observed in difficulty in care and need for help. In these categories, most caregivers with a lack of strength reported that they had difficulties (93.4%) and needed help (81.6%).

As shown in Table 3, a direct correlation existed between perceived lack of physical strength and the ability to perform basic and instrumental activities (*p* < 0.01), the need for caregiver assistance (*p* < 0.01), and the social support perceived (*p* < 0.05) An inverse correlation existed between the conception of the perceived lack of physical strength and the dependence degree of the care receiver (*p* < 0.01).

In the gender analysis, the Spearman correlation coefficient showed a direct relationship among perceived lack of physical strength in both women and men, the ability to perform basic and instrumental activities, and the need for help.

Women had an inverse correlation between the perceived physical strength and the dependency of their care-dependent person. However, this correlation was absent in men.

In the same way, the social support perceived by older women caregivers was inversely correlated with the perception of a lack of physical strength.

## 4. Discussion

The main finding of the present study was that 71% of the older caregivers from rural areas perceived a lack of physical strength. This proportion was similar among male and female older adults, which indicates that both men and women perceived that they have a lack of strength to carry out caregiving tasks. Comparing those with perceived lack of strength and those with enough strength, we can observe how caregivers from the first group need more help, have more difficulties, and show less ability to perform ADL. Furthermore, the dependence level of the person who receives care is also a relevant variable, since more than 80% of the people who had a perceived lack of strength are caring for people with severe or total dependence. Therefore, the perception of strength may be a crucial variable to successfully conduct caregiving.

Caregivers’ health conditions deteriorate with age. As a consequence of normal aging, physical strength and interaction with the social environment decreases [33]. In this sense, our results confirm that the lack of physical strength may determine the need for help in older caregivers from rural areas. Although the prevalence of degenerative muscle mass loss and strength is often higher in older women than in older men [34], there were no differences between the proportion of men reporting lack of strength and the proportion of women with the same perception. Although hypothetical, since most studies reported this result, it is likely that these men measured strength more objectively than women do. They could even have a more absolute perception of strength [35], but when they were asked about the lack of strength to carry out caregiving tasks, these differences were highly reduced.

One of the consequences of sarcopenia [36], an overall loss in skeletal muscle mass that progresses with age, is the decline of gripping force. This fact would support our finding that older caregivers who perceive a lack of physical strength are those who take care of more dependent people and consequently need to develop more tasks and responsibilities where gripping force is crucial. In this regard, older people in primary caregiving roles who have decreased their ability to perform basic and instrumental activities also have a more significant loss of physical strength [33]. Our findings confirm the association between ADL, IADL, and a perceived lack of physical strength.

Due to the physical demands of the care process, caregivers have better scores than non-caregivers on body composition and strength (in both upper and lower limbs) [37]. Nevertheless, they tend to perceive this strength as insufficient to perform some care-related activities [37]. Therefore, strength training interventions in caregivers could increase the satisfaction of the older caregiver in care-related activities. Resistance exercise training can increase muscle mass and strength. Recent evidence suggests that muscle power is an important determinant of functional capacity. Moreover, nutritional interventions, such as the increase of creatine monohydrate, milk-based proteins, and essential fatty acids in the diet, all have biological effects that may potentiate the beneficial effects of exercise in older adults [18,38].

Apart from the physical function variables, cultural factors in the care process are relevant [39,40], since they could condition the care situation perception and its consequences [41,42,43]. In this regard, older adults could see the care process as a moral obligation and may feel guilty if they do not perform the needed functions [44]. This could lead to situations where the caregivers perform tasks that might involve different kind of risks, such as injuries, muscle soreness, anxiety, and falls. Thus, the evaluation and improvement of physical fitness are essential in this population, since they are going to perform those tasks even when their own health is at risk. To do that, a simple test called the International Fitness Scale (IFIS), which has been validated in older adults, can be used to evaluate a person’s perception of their own physical fitness [35]. Through this scale, the potential physical limitations of the caregivers may be identified, and tailored interventions can be conducted.

This study had some limitations. First, we did not use population-based sampling strategies, which limited its generalization as normative values for older caregivers in Spain. However, the socio-demographic characteristics of caregivers were consistent with those reported by large studies previously conducted in Spain [45,46]. Moreover, the physical strength assessment of older caregivers was not objectively assessed, and only the response of the individual’s perception was used. Thus, an in-depth analysis of physical strength requires adding an objective measure of the variable, as well as other variables, such as the weight of the care recipient, the tasks performed, and the instruments or techniques used to mobilize them. In addition, through specific virtual training applications, the caregiver could perceive an increase in strength.

New information and communication technologies (ICTs) have become essential tools in the health sector and an option for caregiver support. Instruments and procedures have been defined that allow the acquisition, treatment, communication, and recording of information in the form of voice, images, and data contained in acoustic, optical, or electromagnetic signals [47]. In general, ICTs are an aid for caregivers who have limited free time, and the use of ICTs would solve the problem of travel, which often limits the caregiver [37,48]. The advantages of ICTs include: (1) the support of the exchange of information; (2) an improved access to medical care; (3) a reduction in costs; and (4) the ability to receive personalized medical care [49,50,51]. In line with our results, future works should study the use of tele-exercise and telemedicine as tools to support older caregivers. In this regard, a previous study showed that tele-medicine had positive effects on muscle strength in older adults [52]. This kind of intervention should be useful in older caregivers, who usually have limited free time. However, the ICT applications used in health services have some limitations among older adults because older adults frequently resist the use of new technologies [53]. In this regard, future studies should investigate the best tool or training method for the acquisition of new knowledge and skills necessary for the use of electronic devices and information systems.

Caregivers require technological tools that allow them to provide better care, and more timely and efficient care for the dependent patient. The applications and services that are currently being developed facilitate the improvement of quality, equality, and access to social and medical care [54].

## 5. Conclusions

In our study, more than 70% of the older caregivers from rural areas perceived they had a lack of strength to conduct their caregiving tasks. Caregivers with a perceived lack of strength need more help, have more difficulties physically caring for others, and show less ability to perform ADL than those without a lack of strength. Furthermore, more than 80% of the people who had a perceived lack of strength are caring for people with severe or total dependence. Therefore, the perception of strength and the characteristics of the cared person may be crucial variables to successfully conduct caregiving tasks.

Older caregivers who have a perception of low social support also perceive a lack of strength to perform care tasks. These findings imply the need to include the perceived physical strength in the comprehensive assessment of older caregivers, in order to identify caregiver’s limitations and needs in their usual care-related activities.

## Figures and Tables

**Table 1:** Sociodemographic variables and comparisons between women and men caregivers.

	Total Sample (*n* = 107)	Women (*n* = 64)	Men (*n* = 43)	Mann–Whitney U or Chi-Square *p*-Value
**Age (years)**				0.008 *
Median (IQR)	78 (8)	76 (9)	79 (7)	
Mean (SD)	76.9 (6.2)	76 (6.3)	78.93 (5.7)	
Range	65–92	65–89	66–92	
**Relationship with the care recipient**				0.177
Husband/wife	90 (84.1%)	51 (79.7%)	39 (90.7%)	
Brother/Sister	10 (9.3%)	8 (12.5%)	2 (4.7%)	
Mother/Father	6 (5.6%)	5 (7.8%)	1 (2.3%)	
Daughter/son	1 (0.9%)	0	1 (2.3%)	
**Previous care experience n (%)**				<0.001 *
Yes	48 (44.9%)	45 (70.3%)	3 (7%)	
No	59 (55.1%)	19 (29.7%)	40 (93%)	
**Care years (years)**				0.291
Median (IQR)	4.5 (7)	4 (7.75)	5 (7)	
Mean (SD)	5.92 (4.59)	5.84 (5.16)	6.03 (3.63)	
Range	0.5–30	0.5–30	1–13	
Care hours (hours)				0.733
Median (IQR)	24 (14)	24 (14)	24 (14)	
Mean (SD)	18.97 (7.86)	19.19 (7.89)	18.65 (7.90)	
Range	2–24	2–24	3–24	
**Difficulty in care n (%)**				0.580
Yes	77 (71.9%)	46 (71.9%)	31 (72.1%)	
No	30 (28.1%)	18 (28.1%)	12 (27.9%)	
**Lack of perceived physical strength n (%)**				0.490
Yes	76 (71.0%)	46 (71.9%)	30 (69.8%)	
No	31 (29.0%)	18 (28.1%)	13 (30.2%)	
**Present doubts about how to take better care n (%)**				0.041 *
Yes	20 (18.7%)	8 (12.5%)	12 (27.9%)	
No	87 (81.3%)	56 (87.5%)	31 (72.º%)	
**Need help n (%)**				0.155
Yes	75 (70.1%)	42 (65.6%)	33 (76.7%)	
No	32 (29.9%)	22 (34.4%)	10 (23.3%)	
**Perceived social support**				0.460
Median (IQR)	45 (16)	44.50 (16.75)	48 (17)	
Mean (SD)	42.41 (12.21)	41.69 (12.65)	43.49 (11.60)	
Range	11–55	11–55	13–55	
**Dependency degree of the care receiver n (%)**				0.078
Moderate dependence	30 (28.0%)	23 (35.9%)	7 (16.3%)	
Severe dependence	26 (24.3%)	13 (20.3%)	13 (30.2%)	
Total dependence	51 (47.7%)	28 (43.8%)	23 (53.5%)	
**Gender of the care receiver n (%)**				<0.001 *
Women	46 (43.0%)	9 (14.1%)	37 (86.0%)	
Men	61 (57.0%)	55 (85.9%)	6 (14.0%)	
**Barthel Index (ADL)**				0.235
Median (IQR)	95 (15)	95 (10)	95 (10)	
Mean (SD)	90.65 (10.64)	90.55 (12.34)	90.81 (7.55)	
Range	45–100	45–100	65–100	
**Lawton and Brody (IADL)**				0.006 *
Median (IQR)	6 (3)	6 (3)	5 (4)	
Mean (SD)	5.39 (2.22)	5.86 (2.11)	4.70 (2.22)	
Range	1–8	1–8	1–8	
**Perceived burden**				0.416
Median (IQR)	47 (28)	44.50 (26.75)	52 (30)	
Mean (SD)	47.78 (15.53)	46.59 (15.17)	49.53 (16.06)	
Range	26–84	26–84	27–83	

* *p*-value < 0.05; SD: Standard Deviation; IQR: Interquartile Range. Barthel: 0–20: total dependence; 21–60: severe dependence; 61–90: moderate dependence; 91–99: light dependence; 100: independent; Lawton and Brody: 0–1: total dependence; 2–3: severe dependence; 4–5: moderate dependence; 6–7: light dependence; 8: independence; Zarit: ≤46: do not perceive burden; 47–55: mild burden perception; ≥56–110: heavy burden perception; questionnaire DUKE-UNC: ≥32: regular support; ˂32 low social support perceived.

**Table 2:** Differences between those who perceived they had a lack of strength and those who did not.

	Lack of Strength (*n* = 76)	No Lack of Strength (*n* = 31)	Mann–Whitney U or Chi-Square *p*-Value
**Age (years)**			0.051
Median (IQR)	79 (9)	76 (8)	
Mean (SD)	77.61 (6.44)	75.16 (5.50)	
Range	65–92	65–85	
**Difficulty in care n (%)**			<0.001 *
Yes	71 (93.4%)	6 (19.4%)	
No	5 (6.6%)	25 (80.6%)	
**Need help n (%)**			<0.001 *
Yes	62 (81.6%)	13 (41.9%)	
No	14 (18.4%)	18 (58.1%)	
**Perceived social support**			0.491
Median (IQR)	45 (16)	49 (22)	
Mean (SD)	42.82 (10.75)	41.42 (15.39)	
Range	11–55	12–55	
**Dependency degree of the care receiver n (%)**			0.008
Moderate dependence	15 (19.7%)	15 (48.4%)	
Severe dependence	19 (25%)	7 (22.6%)	
Total dependence	42 (55.3%)	9 (29.0%)	
**Gender of the care receiver n (%)**			0.531
Women	33 (43.4%)	13 (41.9%)	
Men	43 (56.6%)	18 (58.1%)	
**Barthel Index (ADL)**			<0.001 *
Median (IQR)	90 (10)	100 (5)	
Mean (SD)	88.42 (11.52)	96.13 (4.95)	
Range	45–100	85–100	
**Lawton and Brody (IADL)**			<0.001 *
Median (IQR)	5 (4)	7 (2)	
Mean (SD)	4.88 (2.29)	6.65 (1.43)	
Range	1–8	3–8	

* *p*-value < 0.05; Barthel: 0–20: total dependence; 21–60: severe dependence; 61–90: moderate dependence; 91–99: light dependence; 100: independent; Lawton and Brody: 0–1: total dependence; 2–3: severe dependence; 4–5: moderate dependence; 6–7: light dependence; 8: Independence.

**Table 3:** Relationship of the lack of physical strength perceived.

	Lack of Physical Strength Perceived					
	Total Group		Men		Women	
	r	*p*	r	*p*	r	*p*
**Barthel Index (ADL)**	0.382 **	<0.001	0.410 **	0.006	0.373 **	0.002
**Lawton and Brody (IADL)**	0.370 **	<0.001	0.364 *	0.016	0.437 **	<0.001
**Dependency degree of care receiver**	−0.288 **	0.003	−0.163	0.297	−0.368 **	0.003
**Need help**	0.393 **	<0.001	0.477 **	0.001	0.352 **	0.004
**Perceived social support**	−0.208 *	0.031	0.035	0.825	−0.285 *	0.022
**Perceived burden**	−0.184	0.058	−0.280	0.069	−0.125	0.324

r: Spearman correlation coefficient, ** correlation is significant at 0.01 level, * correlation is significant at 0.05 level.
